# Editorial: Challenges in the prevention of prostate cancer

**DOI:** 10.3389/fonc.2023.1342733

**Published:** 2023-12-05

**Authors:** Valeria Naponelli, Saverio Bettuzzi, Andrea Venerando

**Affiliations:** ^1^ Department of Medicine and Surgery, University of Parma, Plesso Biotecnologico Integrato, Parma, Italy; ^2^ Department of Agricultural, Food, Environmental and Animal Sciences, University of Udine, Udine, Italy

**Keywords:** prostate cancer, risk factors, lifestyle - related disease, prevention, biomarkers

Prostate cancer (PCa) is one of the most common cancers affecting the male population and is a leading cause of cancer-related death in men worldwide (1). A diagnosis of PCa affects the quality of life and well-being of middle-aged and elderly men, imposing a major impact on health care costs.

The clinical features of PCa range from indolent, slow-growing local lesions to highly invasive, rapidly metastasizing tumors (2). Patients with indolent tumor can live with the disease for many years and keep it under control by active surveillance. On the other hand, treatment options for aggressive PCa include surgery, radiation therapy, and chemotherapy (3), but it should not be forgotten that these therapeutic strategies are essential when the tumor is in its early stages, but they become ineffective when the tumor has spread to other body districts.

Discrimination between aggressive and indolent PCa as well as the prediction of the future progression of the disease is the real challenge in clinical practice of PCa management. Currently, pre-treatment risk assessment is based on limited clinical parameters, such as histological grading of needle biopsies (4). The benefit of population-based screening with prostate-specific antigen (PSA), the most used approach to identify at-risk patients, is controversial, mainly because of the high frequency of indolent tumors that dopes the result and may result in an anxious condition for the patient and/or to the initiation of treatments that often do not provide a clear survival benefit (5). Relying on a precise, noninvasive, and effective technique to distinguish between indolent and aggressive PCa and therefore personalized treatment decisions has become a social and medical necessity.

In the past decade, several molecular biomarkers have been proposed as diagnostic tools for the early and noninvasive diagnosis of PCa. In particular, the detection of biomarkers in urine samples represents an excellent alternative screening method and has currently become a hot topic of research. To this end, the study by Wang et al. systematically compared the diagnostic performance of four urinary markers [Progensa Prostate Cancer Antigen 3 (PCA3), SelectMDX, ExoDx Prostate Intelliscore (EPI), Mi-ProstateScore (MIPS)].

Thanks to network meta-analysis, the authors determined that SelectMDX and MIPS may be used as more suitable urinary markers for PCa screening and diagnosis. Although PCa urinary markers have some limitations and require more rigorously design, large-scale, multicenter studies are needed to further explore the advantage of these markers in screening PCa patients. Notwithstanding, they represent a real hope and a starting point to validate a non-invasive and specific method for the identification of invasive PCa at an early stage. In fact, this approach will reduce overdiagnosis of suspected PCa patients, unnecessary needle biopsies and the waste of medical resources.

A therapeutic strategy commonly used in the treatment of benign prostatic hyperplasia involves the use of 5-alpha reductase inhibitors (5-ARI, e.g. dutasteride), which in turn have demonstrated to reduce the risk of developing PCa. Unfortunately, 5-ARI can be associated with significant side effects and their use as PCa chemopreventive molecules should be carefully evaluated. Using data from the REDUCE study, Nguyen et al. have created a comprehensive prediction tool that can simultaneously predict the potential benefits and adverse effects of dutasteride treatment. This prediction tool may help determining the appropriateness of chemoprophylaxis with 5-ARI for men at high risk for PCa development. Notably, the generation of personalized prediction for the potential consequences of dutasteride treatment can allow each patient to make an informed decision whether chemoprophylaxis with a 5-ARI would be beneficial for them. Although the proposed metagram does not predict all relevant outcomes with adequate precision, it provides a starting point for the future rationale and aware use of 5-ARI in PCa prevention.

Given the high incidence of PCa and the increasing trend in Western countries, and the difficulty of an exact diagnosis of the disease and the prediction of its course, prevention can prove to be the most rewarding strategy. Prevention may represent an important approach for reducing the morbidity and mortality of PCa and it is applicable to both early and advanced cancer stages. Knowledge of risk factors, especially those that are modifiable and linked to lifestyle, can provide valuable help in preventing disease and improving or maintaining patients’ health.

The paper by Ziglioli et al. aimed to emphasize the most recent studies related to potential healthy lifestyle factors that influence the development of PCa.

The review confirms some convincing evidence regarding the role of some risk factors, such as overweight or obesity. Central adiposity and ethnicity also influence the possibility of developing PCa. The contribution of smoking does not seem entirely clear, while alcohol affects the prevention of PCa in patients treated with 5-ARI. Although conclusive evidence is still needed, these information could enable the shift from treatment of diagnosed PCa to real efforts in achieving an active systematic prevention.

With a similar aim in elucidating the role of PCa’s risk factors, Suh et al. highlighted that high level of triglycerides represents the most important component of the lipid profile which is strongly correlated with the risk and aggressiveness of PCa. Patients who want to monitor their risk of PCa throughout their entire life may find triglyceride level analysis useful. However, lowering triglyceride levels will need to be studied in the future through randomized controlled trials to confirm their preventive effect.

In conclusion, as demonstrated by the contributions to this Research Topic, it is mandatory to shed light and better managed several etiological factors, both biological, environmental, and lifestyle-correlated, that can either contribute to or affect the initiation and progression of PCa to prevent this intricate and deathly pathology.

Even though groundbreaking scientific discoveries have paved the way for a better therapeutic efficacy by targeting key features of this malignancy, and advances in genetic screening and diagnosis have improved the outlook for patients, PCa still require our attention to achieve optimal results on disease management and prevention, as well as on quality of life and patient survival. A goal that unfortunately is not yet completely accomplished.

## Author contributions

VN: Conceptualization, Writing – original draft, Writing – review & editing. SB: Conceptualization, Writing – original draft, Writing – review & editing. AV: Conceptualization, Writing – original draft, Writing – review & editing.
